# Safety Evaluation for Restorin® NMN, a NAD+ Precursor

**DOI:** 10.3389/fphar.2021.749727

**Published:** 2021-11-11

**Authors:** John Turner, Albert Licollari, Emil Mihalcea, Aimin Tan

**Affiliations:** ^1^ US Scientific Corp, Dover, DE, United States; ^2^ Nucro-Technics, a Division of Vimy Ridge Group LTD, Toronto, ON, Canada

**Keywords:** Restorin® NMN, nicotinamide mononucleotide, NMN, 90-days subchronic oral toxicity, safety assessment, no-observed-adverse-effect-level

## Abstract

NAD+ is an abundant molecule in the body and vital to all living cells. NAD+ levels decline with age, and this decline correlates with age-related diseases. Therefore, sustaining NAD+ levels offers potential benefits to healthspan and longevity. Here we conducted toxicity studies to evaluate the safety of Restorin® NMN, a high purity form of the direct NAD+ precursor, β-nicotinamide mononucleotide (NMN). Based on the preliminary toxicity study and a 14-days repeated dose toxicity study at a higher dose level exposure, Restorin® NMN was administered orally to Sprague-Dawley rats for 91 days followed by a 14-days recovery period. The oral doses of 500, 1,000, and 2000 mg/kg/day were compared. There were no test item-related findings that could be considered adverse events in animals dosed at 500 mg/kg/day. The findings in the Restorin® NMN high dose group (2000 mg/kg/day) were similar to the reference item (Nicotinamide Riboside Chloride) dosed at 1740 mg/kg/day: reduced body weight, reductions in body weight gains, and diminished food consumption. In conclusion, the No-Observed-Adverse-Effect-Level (NOAEL) for Restorin® NMN is 1,000 mg/kg/day in female rats and 500 mg/kg/day in male rats, and the Low-Observed-Adverse-Effect-Level (LOAEL) for Resotrin® NMN is 2000 mg/kg/day.

## Introduction

Nicotinamide adenine dinucleotide (NAD+) was first discovered in 1906 as a component that enhanced the rate of alcohol fermentation in yeast extracts (Harden and Young, 1906) and then later described to have a role in redox reactions (Warburg and Christian, 1936) and as a co-substrate for poly-ADP-ribose polymerases (PARPs) (Chambon et al., 1963) and sirtuins (Imai et al., 2000), a family of NAD+-dependent protein deacetylases (SIRT1–7). It is now well established that NAD+ is a co-factor molecule involved in a myriad of biochemical reactions driving diverse biological processes (Verdin, 2015; Okabe et al., 2019; Hong et al., 2020).

NAD+ synthesis is a highly regulated process, and levels of NAD+ rise and fall depending on food intake, exercise, and the time of day (Rajman et al., 2018). However, NAD+ levels steadily decline with age, resulting in altered metabolism and increased disease susceptibility (Schultz and Sinclair, 2016; Fang et al., 2017; Yaku et al., 2018). NAD+ deficiency is closely associated with the biological aging of cells, tissues, and organs, contributing to the development of various age-related diseases, including type 2 diabetes (T2D), obesity, heart failure, Alzheimer’s disease (AD), and cerebral ischemia (Cantó et al., 2015; Lautrup et al., 2019). Restoration of NAD+ levels in old or diseased animals can promote health and extend lifespan (Mouchiroud et al., 2013; Palmer et al., 2021; Reiten et al., 2021). NAD+-boosting molecules hold the promise of increasing the human body’s resilience to many diseases, thereby extending human healthspan and lifespan.

Over the last decade, a theoretical framework has been synthesized that postulates β-nicotinamide mononucleotide (NMN) as an essential physiological signaling molecule that supports cellular NAD+ concentrations (Imai, 2016; Bolotnikova, 2017). Mammalian cells synthesize NAD+ predominantly through NMN to facilitate the function of enzymes in energy metabolism, DNA repair, cellular aging, and senescence (Yang and Sauve, 2016; Khaidizar et al., 2021). NMN is a bioactive nucleotide naturally formed by the reaction between a phosphate group and a nucleoside containing ribose and nicotinamide (NAM) by nicotinamide phosphoribosyltransferase (NAMPT) (Poddar et al., 2019). NMN is found in various natural foods in low quantities from 0.06 mg/100 g in raw beef to 1.88 mg/100 g in edamame (Mills et al., 2016).

Recent preclinical studies have demonstrated that NMN administration can compensate for NAD+ deficiencies and affect diverse pharmacological activities in various diseases (Yoshino et al., 2011; Gomes et al., 2013; Amjad et al., 2021). Many mouse models of disease, including obesity (Stromsdorfer et al., 2016; Uddin et al., 2016; Uddin et al., 2017), diabetes (Revollo et al., 2007; Caton et al., 2011; Yoshino et al., 2011), cardiovascular disease (Lee et al., 2016; Martin et al., 2017), vascular dysfunction (Tarantini et al., 2019), Alzheimer’s disease (Wang et al., 2016), brain ischemia (Klimova et al., 2020), and kidney disease (Guan et al., 2017), have been subjected to the administration of NMN at a daily dose of 500 mg/kg via intraperitoneal injection. These studies suggest, as an intermediate in NAD+ biosynthesis, NMN is a promising agent to reinforce NAD+ metabolism and alleviate age-related pathologic processes *in vivo*, which has promoted NMN to the stage of clinical trial.

Accordingly, there have been several clinical studies evaluating the safety and efficacy of NMN in humans. A single-arm non-randomized intervention examined the single oral administered of 100, 250, and 500 mg NMN, showing that a single oral administration of NMN was safe and effectively metabolized in healthy men without causing any significant deleterious effects (UMIN000021309) (Irie et al., 2020). Another clinical study (ChiCTR2000035138) administrated three dosages (300, 600, and 1,200 mg) of NMN daily to healthy amateur runners during a 6-weeks exercise training program without any observed obvious adverse symptoms or abnormal ECG (Liao et al., 2021). This study found that the combination of NMN supplementation and exercise further improves the ventilatory threshold even among healthy young and middle-aged people and that the improvement of aerobic capacity is dosage-dependent. A 10-weeks, randomized, placebo-controlled, double-blind trial demonstrated that NMN (250 mg/day) supplementation only increased muscle insulin sensitivity, insulin signaling, and muscle remodeling in women with prediabetes who are overweight or obese (NCT03151239) (Yoshino et al., 2021).

Despite the tremendous research efforts aimed at exploiting the therapeutic potential of NMN to treat metabolic and aging-related diseases in rodents, the clinical and toxicological evidence to support its utility is currently insufficient. Thus, further research is needed to increase the prospects of developing drugs based on NMN. To establish regulatory guidelines for NMN administration, the No-Observed Adverse Effect Level (NOAEL) and Low-Observed Adverse Effect Level (LOAEL) — above which the risk of adverse effects significantly increases—need to be determined. The present work aimed to assess the subacute oral toxicity and subchronic oral toxicity of Restorin® NMN (a high purity synthetic form of NMN) in Sprague-Dawley rats.

## Materials and Methods

### Test Compound

β-Nicotinamide mononucleotide (Restorin® NMN, CAS #1094-61-7) was manufactured to 99.9% purity under GMP conditions. The purity of nicotinamide riboside chloride (CAS #23111-00-4) was measured at 99.16%.

Restorin® dose formulations were prepared by dissolving appropriate amounts of the test item in sterile water for injection. Dose formulation analyses showed that Restorin® NMN was completely soluble in water, and the dose formulations were homogeneous and contained the targeted concentrations of NMN. Stability analyses showed that when Restorin® NMN was dissolved in water, it was stable up to 20 h at room temperature and 108 h at 2–8°C.

The test item was administered orally using a blunt tip gavage needle (16 G) attached to a syringe. Each dose group of animals was provided with a new, commercially available, sterilized syringe and gavage needle. The volume of the test item to be administered to each rat was calculated based on the most recent body weight.

### Test System

The rats were obtained from Charles River Laboratories. LabDiet Certified Rodent Diet (#5002) was provided ad libitum to all animals during the acclimatization and study periods. Municipal water was offered ad libitum throughout the acclimatization and study periods. Animals were deprived of food (but not water) overnight, before collecting blood for clinical pathology evaluations. Restorin® NMN was supplied by Seragon (California, United States). All rats were individually housed and, except for the overnight fast before treatment, provided with food and water ad libitum throughout the study.

#### Pilot Toxicity Study for Dose Determination

A preliminary toxicity study with a very high dose of Restorin® NMN in Sprague-Dawley Rats was conducted to determine the highest possible dose administered to rats based on the maximum feasible concentration of Restorin® NMN of the dosing formulation. The rats were randomized by body weight to two groups (n = 5/sex/group) at the beginning of the study. Either a single dose of vehicle (water) or 5,000 mg/kg of Restorin® NMN was administered orally by gavage.

The test and control items were administered orally daily in the morning for 7 days using a blunt tip gavage needle (16 G) attached to a syringe (Supplementary Appendix Table S1). Animals were observed daily. Body weight and food consumption were measured on Days 1 and 7. On Day 8, animals were euthanized using CO2 overexposure, and gross necropsy examinations were performed.

#### 14-days Safety Study Design

A 14-days repeat dose study with Restorin® NMN in Sprague-Dawley Rats, 35 males (233–343 g) and 35 females (184–246 g) 6–8 weeks at start of dosing, was conducted to determine the dose levels for sub-chronic toxicity experimentation. During the 14-days treatment period, each group was treated by gavage daily with either vehicle (water) or 500, 1,000, 3,000, or 5,000 mg/kg/day of Restorin® NMN (Supplementary Appendix Table S2).

Mortality checks and cage-side clinical evaluations were performed twice daily on all study animals. Detailed clinical examinations were conducted weekly. Observation elements included reactions to treatment, such as changes in the skin, fur, eyes, and mucous membranes. The patterns of respiratory, circulatory, autonomic, central nervous system, and somatomotor activity, as well as behavior were monitored along with any other signs of ill health. Body weights were measured in Week 1 (for randomization), prior to dosing on Day 1, then weekly during treatment, and terminally prior to necropsy (Day 15). The food consumption of each animal was measured weekly. Clinical pathology investigations (hematology, coagulation, clinical chemistry, and urinalysis) were performed for all animals on Day 15 prior to necropsy. Blood samples (∼6 ml) were collected from the abdominal aorta or the heart following anesthesia induced by exposure to isoflurane. Animals were deprived of food (but not water) overnight (approximately 12–18 h), prior to bleeding. Urine was collected from all animals during the last 3 days of the treatment period by placing the animals in metabolic cages for a four-to 6-h period.

#### 91-days Toxicity Study Design

A 91-days repeated oral dose toxicity study with Restorin® NMN in Sprague-Dawley Rats, 65 males (254.0–308.3 g, 55 days old) and 65 females (185.3–242.9 g, 58 days), followed by a 14-days recovery period was conducted in compliance with the Good Laboratory Practices of the OECD4 and the United States Food and Drug Administration5. Restorin® NMN was administered daily by oral gavage to male and female Sprague Dawley rats at doses of 500, 1,000, and 2000 mg/kg/day for 91-days and followed by a 14-days recovery period (Supplementary Appendix Table S3). The results of Restorin® NMN administered at the high dose (2000 mg/kg/day) obtained in this study were compared with an approximate equivalent dose of the reference product (Nicotinamide Riboside Chloride, 1740 mg/kg/day).

In order to verify homogeneity of the test item in dosing formulations of low and high doses, representative samples (0.5 ml in duplicate) were collected from the top, middle, and bottom regions of formulations prepared on Day 1 and the last day of preparation (Day 91). The reference item dosing formulations were sampled similarly on Day 1 and on the last day of preparation (Day 91) 6. In order to verify the concentration of the test item in each dosing formulation, representative samples (0.5 ml in duplicate) were collected from all dose formulations on Day 1 and the last day of preparation (Day 91). The reference item was sampled similarly on Day 1 and the last day of preparation (Day 91). One aliquot of each sample was analyzed under GLP conditions. The backup aliquots were stored frozen (−10 to −25°C). The acceptance criteria for the concentrations of dose formulations were 100 ± 15% of the targeted concentrations.

Male and female rats [strain: Crl: CD® Sprague-Dawley (SD); Charles River] were acclimatized for this study for 14 days. During the acclimatization period, observations for clinical signs and ophthalmoscopy were conducted, and body weights and food consumption were measured. Following the pre-study evaluations, rats were randomized by body weight to control and test groups. The test items were administered orally to rats using low, mid, and high doses daily for 91 days followed by a 14-days recovery period along with control and reference groups.

Clinical signs were recorded at least twice a day during the morning and afternoon observation periods. Detailed clinical examinations were conducted weekly. The body weight of each animal was recorded prior to dosing on Day 1, weekly there after, and terminally prior to necropsy (Day 92 for Main Study animals, Day 106 for Recovery animals). During the recovery period, body weights for Recovery animals were recorded weekly. Food consumption for each rat was recorded weekly during both the treatment and recovery periods. Ophthalmology evaluations were performed for all Main Study and Recovery animals during the last week of dosing.

Clinical pathology investigations (hematology, coagulation, and clinical chemistry) were performed for all Main Study animals at the end of the treatment period on Day 92 and for Recovery animals at the end of the recovery period on Day 106. Full gross pathology was performed on all animals at the end of the respective study periods. Hematology parameters were determined using an ADVIA 120 Hematology system (Siemens Diagnostics). Coagulation parameters were analyzed by a mechanical coagulometric method using a STA Compact Analyzer (Stago Diagnostica). Clinical chemistry parameters were analyzed utilizing a Vitros 350 (Ortho-Clinical Diagnostics). Urinalysis chemical analysis utilized Siemens Multistix® 10 SG. Microscopic examination of the urine sediment was performed using an Olympus BX 50 microscope.

Histopathology was performed on a comprehensive range of tissues from Main Study and Recovery animals in the control and high dose groups as well as target organs in the low and mid-dose groups. During necropsy, the tissues and organs were retained from all animals for histological analysis. Neutral buffered 10% formalin was used for fixation and preservation unless otherwise indicated. With the exception of the animal identification, the tissues listed under Tissue Preservation from Main Study and Recovery animals in the control, high dose, and reference groups as well as tissues with gross lesions were prepared for microscopic examination by embedding in paraffin wax and then sectioning and staining with Hematoxylin and Eosin (H and E). All such tissues were examined microscopically and evaluated histopathologically by a Board-Certified Veterinary Pathologist. Lacrimal glands, optic nerves and parathyroids were examined only if present in routine sections.

### Statistical Analysis

In-life data (dosing, clinical signs, body weights, food consumption, clinical pathology results) were collected using the Ascentos software version 1.3.0 (PDS Inc.). Ophthalmology data, necropsy data, organ weights, and histopathology data were manually entered into the Ascentos software version 1.3.0 (PDS Inc.). Numerical data collected during the study were subjected to the calculation of group means and standard deviations.

Statistical analysis of numerical data was done by PDS built-in statistical functionalities or SigmaStat (SysStat). The data were analyzed for homogeneity of variance and normality. Homogeneous data were analyzed using the Analysis of Variance (ANOVA; *p* ≤ 0.05), and the significance of inter-group differences were analyzed using Duncan’s or other appropriate tests. Heterogeneous data were analyzed using the Kruskal-Wallis test, and the significance of inter-group differences between the control and treated groups were assessed using Dunn’s or other appropriate tests. The Student’s T-test was used for statistical comparison between the test item high dose and reference item dose groups.

## Results

### 7-Day and 14-Day Repeated Dose Toxicity Study of Restorin® NMN in Sprague-Dawley Rats

#### 7-Day High Exposure Dose Toxicity Study

Overall, the rats tolerated the 5,000 mg/kg/day dose well but with some reduction in body weight, body weight gains, and food consumption (Supplementary Appendix Table S4). There were no treatment-related findings at necropsy.

Based on these results, 5,000 mg/kg/day was chosen as the highest dose level for the 14-days repeated dose study.

#### 14-Day Repeated Dose Study

One male dosed at 5,000 mg/kg/day was sacrificed on Day 14 as it was moribund. The clinical signs observed included ptosis, mild piloerection, mild passivity, prostration, dyspnea, and weight loss. One other male dosed at 5,000 mg/kg/day showed mild piloerection and weight loss. The females dosed at 5,000 mg/kg/day showed signs of mild piloerection (5/5), dehydration (4/5), weight loss (4/5), and weakness (1/5). Decreased body weight gains were observed in male rats dosed at 3,000 and 5,000 mg/kg/day, and diminished food consumption was observed at 5,000 mg/kg/day in male rats. Decreased body weight gains and food consumption were observed in females dosed at 5,000 mg/kg/day (Supplementary Appendix Table S5).

Clinical pathology results showed an increase of Alanine aminotransferase (ALT) in both males and females dosed at 3,000 and 5,000 mg/kg/day, an increase of total bilirubin in males dosed at 3,000 and 5,000 mg/kg/day, a reduced urine pH in males and females dosed at 5,000 mg/kg/day and an increase in urine crystals in males and females dosed at 3,000 mg/kg/day (3/5 and 1/5, respectively) and also in males and females dosed at 5,000 mg/kg/day (3/5 and 2/5, respectively).

Differences in group mean absolute weights were decreased for body weight and spleen in males dosed at 3,000 mg/kg/day (Group 4-M) and males dosed at 5,000 mg/kg/day (Group 5-M), decreased for the females dosed at 5,000 mg/kg/day (Group 5-F), increased for kidneys in males dosed at 5,000 mg/kg/day and decreased for pituitary and thymus for the females dosed at 5,000 mg/kg/day (Group 5-F) compared with the vehicle control values.

Increases in relative organ weights between control and treated groups were observed in the brain and kidneys of females treated at 5,000 mg/kg/day (Group 5-F) and for kidneys in males treated at 5,000 mg/kg/day (Group 5-M). A decrease in relative weights for the pituitary gland and thymus was observed in animals treated at 5,000 mg/kg/day (Group 5-M and 5-F).

Histopathological findings were noted at the end of the treatment period in multiple organs in animals treated at 3,000 and 5,000 mg/kg/day. These findings consisted of renal tubule necrosis, mineralization, crystal aggregates, and obstructive nephropathy in male and female rats. These regimens also caused mucosal erosion with mucosal hyperplasia in the stomach, periacinar necrosis in the liver, lymphoid atrophy in the thymus and spleen, and hyaline droplet accumulation in the renal proximal tubules (males only).

Restorin® NMN dosed at 5,000 mg/kg/day for 14 days was associated with mortality and clinical signs that included eye ptosis, moderate dyspnea, mild piloerection, and weight loss. Restorin® NMN at 3,000 mg/kg/day and 5,000 mg/kg/day resulted in reduced body weight gain and reduced food consumption. No clinical signs were observed in the rats dosed at 3,000 mg/kg/day. These regimens also caused erosion with hyperplasia in the glandular mucosa of the stomach, periacinar necrosis in the liver, lymphoid atrophy in the thymus and spleen, and hyaline droplet accumulation in the renal proximal tubules (males only), clinical and anatomical pathology findings, and neoplastic histopathological changes that were considered dose-related in incidence compared to the vehicle control group.

Restorin® NMN doses of 500 and 1,000 mg/kg/day dosed orally for 14 days were not associated with any test item adverse events.

### A 91-Day Repeated Oral Dose Toxicity Study With Restorin® NMN in Sprague-Dawley Rats Followed by a 14-Day Recovery Period

#### Mortality and Clinical Observations

All animals completed the 91-days treatment period. Recovery animals completed the 14-days recovery period. There were no clinical observations that were considered to be related to treatment with either test or reference items.

#### Body Weights, Body Weight Changes, and Food Consumption

As the treatment period progressed, there were dose-dependent reductions of the overall body weight and body weight gains in males and females. In males, there were statistically significant decreases in mean body weights of mid-dose males on Days 71-91 (*p* < 0.05) compared to the control group (Supplementary Appendix Table S6). In the high dose males and reference dose males, there were statistically significant decreases in mean body weights on Days 15-91 (*p* < 0.01), compared to the control group. There were also statistically significant decreases in mean body weight gains in these groups throughout the treatment period compared to the control group. From Days 1–91, there was a dose-related reduction in body weight gain, with the high dose group and reference dose group significantly lower (*p* < 0.01) than the control group. These findings were also coupled with significant decreases in food consumption week by week in the high dose males throughout the study period along with an overall significant decrease in food consumption in the reference dose males.

In the high dose females, there were statistically significant decreases in mean body weights on Days 57–91 (*p* < 0.05) and in the reference dose from Days 85–91 compared to the control group (Supplementary Appendix Table S7). There were also statistically significant (*p* < 0.01) decreases in mean body weight gains in the high dose group sporadically during the treatment period. From Days 1–91, there was a dose-related reduction in body weight gain, with the high dose group and reference dose group significantly lower (*p* < 0.01) than the control group.

Throughout the recovery period, the high dose male and female groups still showed statistically significant (*p* < 0.01 and *p* < 0.05) lower body weight on Days 92–105 for males and females. However, statistically significant increases (*p* < 0.01) in mean body weight gains in the high dose and reference male groups compared to the control group indicated reversibility of the body weight reduction. In females, there were no statistically significant changes in body weight gains noted in the high dose females throughout the recovery period, indicating some recovery of the decreased weight gains at the end of the 14-days recovery period.

#### Clinical Pathology

On a few occasions, individual values for hematology and clinical chemistry parameters were slightly outside the established normal reference ranges. However, these out-of-range (OR) values were observed in the control group and therefore were considered incidental, not associated with other findings, and of no toxicological significance. A list of the methods, abbreviations, and units used for the clinical pathology parameters tested is included in the Appendix.

##### Hematology Data

At the end of the treatment period, there were two test and reference item-related findings in hematology parameters (Supplementary Appendix Table S8). There was slightly higher (*p* < 0.01) hemoglobin in the high dose (2000 mg/kg/day) male group relative to the control group. A similar high concentration of hemoglobin was observed in the group dosed with the reference product. In addition, the neutrophil count and proportion to WBC were higher, while the lymphocyte proportion was lower in the high dose males relative to the control group. Similar findings were present in the group treated with the reference product. There were no test or reference item differences in hematology parameters at the end of the recovery period.

A few statistically significant changes were seen in hematology parameters when comparing the reference dose to the high dose in the female rats at the end of the treatment period (Supplementary Appendix Table S8). These differences between the high dose test item and reference item NRC were considered not physiologically significant. There were no other statistically significant hematology parameters at the end of the treatment and the recovery period.

##### Clinical Chemistry

At the end of the treatment period, there were various statistically significant test and reference item-related findings in clinical chemistry parameters (Supplementary Appendix Table S9). There was an increase in alkaline phosphatase (ALKP) in males and females in the test item high dose and the reference groups compared to the control group. The magnitude of changes was minimal, and while they may be related to the test item, this finding was not associated with histopathological changes. There was also an increase in alanine transaminase (ALT) in mid and high dose males dosed with the test item and reference item and females in the high dose group compared to the control group. The magnitude of changes in ALT was less than two-fold compared to the control group and was not associated with significant liver changes. Lastly, the cholesterol was lower in males of all treated groups relative to the control group, but the magnitude of change was minimal and not physiologically significant.

The statistically significant changes in clinical chemistry parameters between the high-dose test item group and the reference group were minor and of no physiological significance (Supplementary Appendix Table S10). There were no other statistically significant clinical chemistry parameters at the end of the treatment and the recovery period.

##### Urinalysis Data

Administration of a low, mid, or high dose of the test item or the reference item was associated with urine acidification in both males and females at the end of the treatment period. The test item administration was also associated with reduced urine volume and increased specific gravity in mid and high dose-treated males and females and animals treated with the reference item. The reduction in urine pH in treatment groups likely related to renal excretion of the test and reference items but did not necessarily indicate renal dysfunction or injury. At the end of the recovery period, these changes were no longer present. Slightly higher specific gravity in the high dose-treated females at the end of the recovery period is considered incidental. There were no significant changes in the microscopic urinalysis results for any group.

#### Gross Pathology

Restorin® NMN and Nicotinamide Riboside Chloride (NRC), administered orally in Sprague-Dawley rats, did not produce any gross lesions related to test item treatment. Only two gross lesions, alopecia in the skin, and hemorrhage/nodular lesions in the ear were observed in more than three animals. Both of these lesions were in control and treatment groups in both male and female animals, and their distribution does not suggest a treatment effect.

#### Organ Weights

The biologically and statistically significant differences from the control group were found for absolute organ weights and organ to body weight ratios of treatment groups compared to controls at the end of the treatment period (Day 92). At the end of the treatment period, there were several statistically significant changes in organ weights compared to the control group (Supplementary Appendix Table S11 and Supplementary Appendix Table S12). None of the differences were significant when expressed as a percentage of brain weight. Some of these differences above relate to the reduction in body weight in the high-dose test item and reference item male groups (Groups 4-M and 5-M). Other statistically significant findings in organ weights were considered incidental and not associated with histopathological findings, and therefore not test item-related. There were no microscopic findings to explain the differences in organ weights for brain, heart, spleen, thymus, ovaries, and testes. There were no significant differences in organ weights when comparing Groups 4 (Restorin® 2000 mg/kg/day) and 5 (NRC 1740 mg/kg/day) at Day 92 or Day 106, with the exception of decreased lung to body weight ratio in Group 5 males compared to Group 4 males on Day 106 (−7.3%).

Increases in kidney weights were the most consistent dose-related changes at Day 92 but at Day 106, these were not significantly different from the Control Group. Small areas of zonal hypertrophy were observed in the livers of some animals in the high-dose NMN and NRC treated groups (Groups 4 and 5). At the end of the recovery period, the mean liver to body weight ratio in the NRC-treated male group was significantly higher (11.1%; *p* < 0.05), and the mean pituitary weight in the NRC-treated male group was statistically significantly lower (−12.6%; *p* < 0.05) compared to the control group. The mean thymus to body weight ratio was greater and statistically more significant (35.1%; *p* < 0.05) in the NRC-treated female group compared to the control group. In high dose males and females, statistically significant increases in the organ/body weight ratios observed for the liver and thymus were due to the lower body weights of the high dose animals.

#### Histopathology

Some possible adverse microscopic findings associated with treatment included increased numbers of round eosinophilic inclusions (hyaline droplets) observed in the proximal tubules of some male rats in the test item high dose and the reference item group. Several animals in the high dose group and reference item group had early stages of chronic progressive nephropathy (CPN), a spontaneous rat-specific disease. Various histological findings described in the histopathology report were either known background conditions in Sprague-Dawley rats or sporadic and incidental findings of negligible clinical or toxicological significance. The frequency and severity of these changes were not related to the test and reference item treatment.

## Discussion

There are many ways of restoring NAD+ level depletion caused by aging or other diseases, including improving NAMPT expression, providing NAD+ precursors, or inhibiting the NAD+ consuming enzymatic activities of PARP (Bai et al., 2011; Scheibye-Knudsen et al., 2014), CD38, and SARM1. Currently, supplementation with NMN is considered a viable and highly efficient strategy of increasing NAD+ levels. Accordingly, NMN has shown safety and promise in conditions of metabolic stress and aging in a variety of animal models. To date, there is minimal available data on the safety parameters of NMN that could allow determination of its No-Observed Adverse Effect Level (NOAEL) and no authoritative or regulatory body to establish the tolerable upper intake level (UL) for humans, which represents the threshold above which the risk of adverse effects significantly increases.

In this study, the test item-related findings in the animals exposed at 1,000 mg/kg/day included a slight reduction in the body weight in male rats close to the end of treatment period and a mild increase in ALT. There were no test item-related findings that could be considered adverse events in animals dosed at 500 mg/kg/day. Therefore, under the conditions of this study, the No-Observed-Adverse-Effect-Level (NOAEL) for Restorin® NMN is 1,000 mg/kg/day in female rats and 500 mg/kg/day in male rats. The Low-Observed-Adverse-Effect-Level (LOAEL) for Restorin® NMN is 2000 mg/kg/day. Restorin® NMN administered to Sprague-Dawley rats by oral gavage daily for 91 days in the dose range of 500–2000 mg/kg/day resulted in adverse test item-related findings observed at the dose level 2000 mg/kg/day and included reduced body weight, reductions in body weight gains, diminished food consumption, and increased ALT levels for both male and female rats. Similarly, reductions in body weight and food consumption were observed in the reference item NRC administered at a dose of 1740 mg/kg/day. ALT was higher in both male and female high Restorin® NMN dose-treated groups and the mid Restorin® NMN dose-treated group compared to the control group. The magnitude of changes was very small, and while they may be related to Restorin® NMN, they do not indicate physiologically significant liver disease.

Male-specific test item-related findings that were observed at Restorin® NMN dose level of 2000 mg/kg/day included increased hemoglobin, neutrophils, and ALKP levels and decreased urine pH. The changes in male levels of hemoglobin, which was also observed in the group dosed with the reference product NR, could be due to reduced water intake, leading to mild volume contraction and increased hemoglobin. The increase in neutrophil counts may reflect altered circulating glucocorticoids or catecholamines, but the change was not physiologically significant (Ince et al., 2019). ALKP was higher in high Restorin® NMN dose-treated males and NRC treated males; however, the magnitude of changes in ALKP was small and ALKP values were less than 2-fold and were not associated with liver changes. The reduction in urine pH in high Restorin® NMN dose-treated males and NRC treated groups likely related to renal excretion of the test and reference agents but does not necessarily indicate renal dysfunction or injury.

In addition, for Restorin® NMN high dose and respective equivalent dose of the reference item NRC, biologically and statistically significant differences in the organ weights at the end of the treatment period included increased kidney weights in males when compared to control. The increased kidney weights correlated with the increased hyaline droplets in the proximal renal tubules. This finding in kidneys is a well-recognized response of male rats to various lipophilic xenobiotics that bind to alpha-2u-globulin, a small pheromone-binding plasma protein that undergoes glomerular filtration, reabsorption and lysosomal degradation in the proximal tubules in male rats. The response, termed alpha-2u-globulin nephropathy, is a male-rat specific response, because neither female rats nor humans produce alpha-2u-globulin (Frazier et al., 2012). The frequency and grade of the hyaline droplet responses were similar in male rats given Restorin® NMN or the reference item NRC at the end of the 91-days treatment period and after the 14-days recovery period. Serum biochemistry indicators of renal dysfunction (creatinine, BUN, albumin, phosphorus) were within reference ranges and were not significantly and substantially different from control group.

These experiments were performed using injection of Restorin® NMN. The gut-expressed multi-spanning membrane protein Slc12a8, previously annotated as a Na+/K + Cl− transporter, has been proposed to be a specific transporter of NMN (Grozio et al., 2019). Indeed, Mills and colleagues demonstrated in 2016 that transport of NMN from the gut to the circulation and then to tissues occurred within 10 min in mice. Remarkably, the function of the Slc12a8 NMN transporter becomes crucial in aged individuals compared to young ones. In response to significant decreases in NAD+ levels, the aged ileum upregulates Slc12a8 expression in attempts to maintain its NAD+ levels. When enough NMN is supplied, this feedback system can function adequately to maintain levels of NAD+ comparable to those in young ilea. Therefore, increasing NMN availability or stimulating the function of the NMN transporter could effectively counteract age-associated NAD+ decline in the aged small intestine. Yoshino and colleagues also showed in 2011 that bolus intraperitoneal administration of NMN (500 mg/kg/day) increased tissue NMN and NAD+ levels within 15 min in the liver, pancreas, and white adipose tissue in regular chow-fed wild-type mice. The expression of Slc12a8 in the gut suggests that NMN could be made bioavailable through oral delivery to make long-term NMN administration more accessible. Indeed, Mills and colleagues showed that NMN administered through drinking water and oral gavage (300 mg/kg/day) to mice is quickly absorbed, efficiently transported into blood circulation, and immediately converted to NAD+ in major metabolic tissues.

To our knowledge, tests of the safety and oral availability of NMN in humans are lacking. Although the safety of long-term NMN administration in humans remains unclear, clinical trials have been launched to investigate the safety and benefits of NMN. The NAD+ production and consumption pathways including NMN are essential for a more precise understanding of age-related pathological processes such as diabetes, ischemia-reperfusion injury, heart failure, Alzheimer’s disease, and retinal degeneration. It will be of great interest to translate our study from mice to humans and examine whether NMN is safe either when injected intravenously or through oral administration.

## Data Availability

The original contributions presented in the study are included in the article/Supplementary Material, further inquiries can be directed to the corresponding author.

## Glossary

ALB: Albumin
ALKP: alkaline phosphatase
ALT: serum alanine aminotransferase
ANOVA: analysis of variance
Ca: Calcium
CCAC: Canadian Council on Animal Care
CFR: Code of Federal Regulations
CHOL: Cholesterol
Cl: Chloride
CREA: Creatinine
F: Female
G: Gauge
g: Grams
HEMO: Hemoglobin
hr: Hour
ID: Identification
K: Potassium
kg: Kilogram
L: Liter
LDH: lactate dehydrogenase
LUC: large unstained cells
LYM: Lymphocytes
M: Male
MCV: mean corpuscular volume
mL: Milliliter
mmol: Millimolar
MO: Monocytes
n: number of determinations
N/A: not applicable
Na: Sodium
Neg.: Negative
NEU: Neutrophils
NMN: nicotinamide mononucleotide
No.: Number
NOAEL: no-observed-adverse-effect-level
NRC: Nicotinamide Riboside Chloride
OECD: Organization for Economic Cooperation and Development
OR: out of range
Phos: Phosphorus
PLAT: Platelets
SD: standard deviation
TP: total protein
WBC: white blood cell count
