# Supplementary material for: Safety Evaluation for Restorin® NMN, a NAD+ Precursor
Source: Front Pharmacol. 2021 Nov 11;12:749727. doi: 10.3389/fphar.2021.749727 (PMC8632654; doi:10.3389/fphar.2021.749727)
Supplement: Supplementary file 1 [file Table1.DOCX]

**Appendex I Tables**

***Table 1. Pilot Toxicity Study Outline***

| **Group** | **Dose (mg/kg/day)** | **Males** | **Females** |
| --- | --- | --- | --- |
| Control Vehicle (WFI*) | 0 | 5 | 5 |
| High Dose | 5000 | 5 | 5 |

*Sterile water for injection (WFI)

***Table 2. 14-day Safety Study Outline***

| **Group** | **Treatment Designation** | **Dose (mg/kg/day)** | **Concentration in Dose Formulation (mg/mL)** | **Dose Volume (mL/kg)** | **Main Study Animals (Necropsy Day 15)** | |
| --- | --- | --- | --- | --- | --- | --- |
|  |  |  |  |  | **Male** | **Female** |
| **1** | Control (water) | 0 | 0 | 10 | 5 | 5 |
| **2** | Low Dose | 500 | 50 | 10 | 5 | 5 |
| **3** | Mild Dose | 1000 | 100 | 10 | 5 | 5 |
| **4** | High Dose | 3000 | 300 | 10 | 5 | 5 |
| **5** | Very High Dose | 5000 | 500 | 10 | 5 | 5 |

***Table 3. 91-Day Toxicity Study Outline***

| **Group #** | **Group** | **Dose** | **Dose Volume (mL/kg)** | **Dosing Solution Conc. (µg/mL)** | **Main Study^1^** | | **Recovery^2^** | | **Frequency of Dosing** |
| --- | --- | --- | --- | --- | --- | --- | --- | --- | --- |
|  |  | **(mg/kg)** |  |  | **Male** | **Female** | **Male** | **Female** |  |
| 1 | Control | 0 | 10 | 0 | 10 | 10 | 5 | 5 | Daily for 91 days |
| 2 | Low Dose | 500 | 10 | 50 | 10 | 10 | - | - |  |
| 3 | Mid Dose | 1000 | 10 | 100 | 10 | 10 | - | - |  |
| 4 | High Dose | 2000 | 10 | 200 | 10 | 10 | 5 | 5 |  |
| 5 | Reference^3^ | 1740 | 10 | 174 | 10 | 10 | 5 | 5 |  |

***Table 4 Body Weight and Food Consumption in Pilot Safety Study***

|  | **Group** | **Mean Body Weight (g)** | | **Mean Body Weight Change (g)** | **Mean Food Consumption (g)** |
| --- | --- | --- | --- | --- | --- |
|  |  | **Day 1** | **Day 7** |  |  |
| M | *Control* | 296.3 | 337.0 | +40.8 | 157.1 |
|  | *5000 mg/kg* | 253.7 | **250.2**** | **-3.5**** | **98.0**** |
| F | *Control* | 193.6 | 206.0 | +7.5 | 90.3 |
|  | *5000 mg/kg* | 201.1 | 202.2 | +3.8 | **71.2**** |

*** significantly different from control (p < 0.01)*

***Table 5 Summary of Body Weight and Food Consumption in 14-Day Safety Study***

| **Sex** | **Group** | **Mean Body Weight (g) ± S.D.** | | | **Mean Body**  **Weight**  **Change (g) ± S.D.**  **Day 1-14** | **Mean Food**  **Consumption**  **(g) ± S.D. Day 1-14** |
| --- | --- | --- | --- | --- | --- | --- |
|  |  | **Day 1** | **Day 8** | **Day 14** |  |  |
| Males | Control | 294.5 ± 14.5 | 346.0 ± 24.2 | 390.8 ± 28.3 | 96.3 ± 14.0 | 360.1 ± 32.5 |
|  | 500 mg/kg | 294.4 ± 18.4 | 338.5 ± 20.2 | 373.1 ± 21.6 | 78.7 ± 13.2 | 352.2 ± 25.4 |
|  | 1000 mg/kg | 297.0 ± 24.5 | 339.6 ± 31.6 | 378.7 ± 37.2 | 81.7 ± 13.9 | 372.2 ± 31.4 |
|  | 3000 mg/kg | 291.7 ± 13.8 | 316.6 ± 14.6 | 341.6 ± 21.0 | **49.8* ± 10.7** | 337.3 ± 66.0 |
|  | 5000 mg/kg | 292.5 ± 16.2 | 309.2 ± 23.0 | 315.5 ± 48.1 | **23.1** ± 49.0** | **273.3* ± 40.9** |
| Females | Control | 203.9 ± 11.1 | 223.8 ± 7.2 | 239.8 ± 12.7 | 36.0 ± 6.0 | 240.6 ± 15.2 |
|  | 500 mg/kg | 212.2 ± 9.5 | 231.9 ± 13.3 | 255.1 ± 10.8 | 42.9 ± 8.3 | 279.6 ± 33.3 |
|  | 1000 mg/kg | 215.1 ± 12.2 | 229.1 ± 10.8 | 254.0 ± 12.8 | 38.9 ± 13.7 | 254.4 ± 20.3 |
|  | 3000 mg/kg | 217.1 ± 17.3 | 230.6 ± 19.1 | 251.9 ± 19.5 | 34.8 ± 8.0 | 234.3 ± 23.4 |
|  | 5000 mg/kg | 212.4 ± 9.5 | 198.5 ± 9.4 | 219.5 ± 24.4 | **7.1** ± 22.7** | **157.7** ± 49.6** |

** Statistically significant from control (p < 0.05)*

*** Statistically significant from control (p < 0.01)*

***Table 6.*** ***Summary of Body Weights for Males – Treatment Period***

| **Mean Body Weight (g) ± SD** | **1-M** | **2-M** | **3-M** | **4-M** | **5-M** |
| --- | --- | --- | --- | --- | --- |
| D1 | 289.5 ± 12.4 | 283.4 ± 15.9 | 286.4 ± 11.7 | 287.0 ± 12.8 | 286.5 ± 13.3 |
| D22 | 444.3 ± 17.2 | 443.6 ± 27.9 | 423.4 ± 29.2 | 411.9 ± 27.2** | 410.2 ± 25.0** |
| D43 | 542.1 ± 25.3 | 543.7 ± 44.2 | 512.8 ± 41.2 | 485.2 ± 40.2** | 490.2 ± 35.7** |
| D64 | 618.5 ± 31.4 | 616.2 ± 45.7 | 579.5 ± 47.3 | 535.9 ± 50.1** | 540.8 ± 43.9** |
| D85 | 673.8 ± 46.7 | 674.5 ± 52.1 | 618.0 ± 52.1* | 567.9 ± 54.9** | 577.6 ±56.1** |
| D91 | 679.4 ± 46.1 | 681.6 ± 53.3 | 623.8 ± 52.3* | 572.4 ± 56.5** | 583.3 ± 54.7** |
| D1-91 | 389.9 ± 47.4 | 398.2 ± 45.7 | 337.4 ± 42.7 | 285.4 ± 49.7** | 296.8 ± 50.1** |

** Significantly different from control (p < 0.05)*

*** Significantly different from control (p < 0.01)*

***Table 7.*** ***Summary of Body Weights for Females – Treatment Period***

| **Mean Body Weight (g) ± SD** | **1-F** | **2-F** | **3-F** | **4-F** | **5-F** |
| --- | --- | --- | --- | --- | --- |
| D1 | 208.0 ± 15.7 | 209.8 ± 13.8 | 209.4 ± 14.4 | 211.7 ± 13.7 | 210.0 ± 12.1 |
| D22 | 270.7 ± 24.6 | 268.4 ± 15.8 | 261.3 ± 22.5 | 253.3 ± 18.4 | 256.0 ± 20.7 |
| D43 | 300.7 ± 28.2 | 302.1 ± 23.6 | 292.6 ± 25.3 | 281.1 ± 18.7 | 283.3 ± 27.6 |
| D64 | 329.6 ± 33.3 | 329.2 ± 24.7 | 317.2 ± 30.2 | 299.2 ± 21.0* | 306.2 ± 32.5 |
| D85 | 351.5 ± 41.5 | 348.2 ± 24.7 | 333.5 ± 32.8 | 314.9 ± 21.2* | 319.4 ± 39.1* |
| D91 | 354.8 ± 42.5 | 351.9 ± 24.8 | 338.5 ± 32.8 | 317.8 ± 22.4* | 321.5 ± 39.4* |
| D1-91 | 146.8 ± 32.8 | 142.1 ± 14.2 | 129.1 ± 22.6 | 106.0 ± 16.8** | 111.5 ± 31.0** |

** Significantly different from control (p < 0.05)*

*** Significantly different from control (p < 0.01)*

***Table 8. Hematology Parameters – Percentage Changes from the High Dose Group***

| **Time point** | **Parameter** | **Males** | | **Females** | |
| --- | --- | --- | --- | --- | --- |
|  |  | **High Dose** | **Reference Dose** | **High Dose** | **Reference Dose** |
| End of Treatment | PLAT [x10e9/L] | 638 | -2.40% | 809 | **-25.3%*** |
|  | WBC [x10e9/L] | 10.46 | 17.90% | 8.06 | **-34.7%*** |
|  | LYM [x10e9/L] | 8.11 | 14.80% | 6.76 | **-34.9%*** |
|  | EOS [x10e9/L] | 0.1 | 20.00% | 0.09 | **-33.3%*** |

** Significantly different from control (p < 0.05)*

***Table 9. Clinical Chemistry Parameters - Percentage Changes from Controls***

| **Time point** | **Parameter** | **Males** | | | | | **Females** | | | | |
| --- | --- | --- | --- | --- | --- | --- | --- | --- | --- | --- | --- |
|  |  | **Control** | **Low Dose** | **Mid Dose** | **High Dose** | **Reference Dose** | **Control** | **Low Dose** | **Mid Dose** | **High Dose** | **Refer. Dose** |
| End of Treat. | ALB [g/L] | 30 | -3.30% | 0.00% | **6.7%*** | 6.70% | 36 | **-11.1%*** | -5.60% | -5.60% | 0.00% |
|  | ALKP [U/L] | 106 | 20.80% | 22.60% | **56.6%**** | **41.5%**** | 52 | 1.90% | 13.50% | **94.2%**** | **32.7%*** |
|  | BUN [mmol/L] | 5.5 | -1.80% | -5.50% | 9.10% | **20.0%*** | 5.4 | **-29.6%**** | **-20.4%**** | -11.10% | -7.40% |
|  | Cl [mmol/L] | 102 | -1.00% | -1.00% | -1.00% | **-2.0%**** | 103 | 0.00% | 1.00% | **3.9%**** | 1.90% |
|  | CREA [μmol/L] | 34 | -2.90% | -8.80% | -8.80% | -11.80% | 32 | -3.10% | -9.40% | **-12.5%*** | **-12.5%*** |
|  | LDH [U/L] | 3,393 | -4.00% | -32.40% | **-36.5%*** | -25.70% | 2,540 | 22.20% | 0.50% | -19.90% | -18.00% |
|  | Phos [mmol/L] | 2.09 | 1.00% | **-8.1%*** | **-12.0%**** | -1.90% | 1.9 | 3.20% | 1.10% | -5.30% | 0.00% |
|  | TP [g/L] | 57 | -1.80% | 1.80% | **7.0%**** | 5.30% | 63 | -6.30% | -1.60% | -3.20% | 0.00% |
|  | ALT [U/L] | 46 | 37.00% | **47.8%*** | **71.7%**** | **93.5%**** | 59 | -16.90% | -8.50% | **30.5%*** | 13.60% |
|  | Na [mmol/L] | 139 | 0.70% | 0.70% | 0.00% | 0.00% | 141 | 0.70% | 1.40% | **2.8%**** | **2.1%**** |
|  | CHOL [mmol/L] | 1.85 | **-25.4%**** | **-31.9%**** | **-19.5%*** | **-20.0%*** | 1.95 | **-27.7%**** | -16.90% | -7.70% | -11.80% |
| Recovery | Ca [mmol/L] | 2.43 | - | - | 0.80% | **5.3%*** | 2.56 | - | - | -2.00% | -1.60% |
|  | CHOL [mmol/L] | 1.71 | - | - | -9.40% | 22.80% | 2.27 | - | - | -18.50% | **-22.5%*** |

** Significantly different from control (p < 0.05)*

*** Significantly different from control (p < 0.01)*

*- no animals for this group*

***Table 10. Clinical Chemistry Parameters - Percentage Changes from Controls***

| **Time point** | **Parameter** | **Males** | | **Females** | |
| --- | --- | --- | --- | --- | --- |
|  |  | **High Dose** | **Reference Dose** | **High Dose** | **Reference Dose** |
| End of Treatment | ALKP [U/L] | 166 | -9.60% | 101 | **-31.7%*** |
|  | Cl [mmol/L] | 101 | -1.00% | 107 | **-1.9%*** |
|  | Phos [mmol/L] | 1.84 | **11.4%*** | 1.8 | 5.60% |
|  | Na [mmol/L] | 139 | 0.00% | 145 | **-0.7%*** |
| Recovery | BUN [mmol/L] | 5 | **22.0%*** | 5.3 | -9.40% |
|  | Ca [mmol/L] | 2.45 | **4.5%*** | 2.51 | 0.40% |
|  | CHOL [mmol/L] | 1.55 | **35.5%*** | 1.85 | -4.90% |
|  | Na [mmol/L] | 139 | 1.40% | 139 | **2.2%**** |

# Table 11. Organ Weights and Organ Weight to Body/Brain Weight Ratio at the End of the Treatment Period – Percentage Change from Controls

| *Differences in absolute organ weights compared to control group* | |
| --- | --- |
| Brain | Decreased in Group 4-F |
| Kidneys | Increased in Groups 4-M and 5-M |
| Liver | Increased in Groups 4-M and 5-M |
| Spleen | Reduced in Group 4-M |
| Thymus | Reduced in Group 5-M |
|  | |
| *Differences in organ weights relative to body weight compared to control group* | |
| Brain | Increased in Groups 4-M and 5-M |
| Heart | Increased in Groups 4-M and 5-M |
| Kidneys | Increased in Groups 3-M, 4-M, 4-F, 5-M, and 5-F |
| Liver | Increased in Groups 3-F, 4-M, 4-F, 5-M, and 5-F |
| Ovaries | Increased in Groups 4-F and 5-F |
| Testes | Increased in Group 5-M |

# Table 12. Organ Weights and Organ Weight to Body/Brain Weight Ratio at the End of the Treatment Period – Percentage Change from Controls

| **Organ** | | **Males** | | | | | | | | **Females** | | | | | | | | | |
| --- | --- | --- | --- | --- | --- | --- | --- | --- | --- | --- | --- | --- | --- | --- | --- | --- | --- | --- | --- |
|  |  | **Control^+^** | **Low Dose^+^** | **Mid Dose^+^** | **High Dose^+^** | | **Reference Dose^+^** | | **Control^+^** | | **Low Dose^+^** | | **Mid Dose^+^** | | **High Dose^+^** | | **Reference Dose^+^** | |  |
| final body weight | | 633.2 g | 2.30% | -7.50% | | **-14.3%**** | | **-15.8%**** | | 320.0 g | | 0.40% | | -3.50% | | -9.00% | | -11.00% | |
| brain | *mean weight* | 2.164 g | -2.07% | -3.72% | -3.03% | | -3.91% | | 1.97% | | -1.77% | | -2.03% | | **-6.10%*** | | -4.20% | |  |
|  | *organ/body* | 0.34% | -4.16% | 4.24% | **+13.45%**** | | **+14.76%**** | | 0.63% | | -2.90% | | 1.43% | | 2.39% | | 6.91% | |  |
| heart | *mean weight* | 1.810 g | 2.66% | -1.64% | -5.56% | | -4.14% | | 1.084 g | | 0.76% | | 0.19% | | -5.36% | | -1.99% | |  |
|  | *organ/body* | 0.29% | 0.22% | 6.08% | **+10.12%*** | | **+11.30%**** | | 0.34% | | 1.96% | | 5.64% | | 5.14% | | 7.54% | |  |
|  | *organ/brain* | 83.65% | 4.68% | 2.11% | -2.38% | | -0.27% | | 54.80% | | 2.98% | | 2.65% | | 1.23% | | 2.68% | |  |
| spleen | *mean weight* | 1.041 g | -6.37% | -13.83% | **-18.06%**** | | -11.93% | | 0.604 g | | -0.61% | | -0.83% | | -3.06% | | -9.39% | |  |
|  | *organ/body* | 0.17% | -8.60% | -7.16% | -5.02% | | 0.03% | | 0.19% | | -3.14% | | -3.86% | | 2.34% | | -2.33% | |  |
|  | *organ/brain* | 48.15% | -4.52% | -10.57% | -15.49% | | -8.51% | | 30.49% | | 1.68% | | 1.43% | | 4.06% | | -4.67% | |  |
| thymus | *mean weight* | 0.547 g | 2.00% | -16.11% | -26.41% | | **-35.25%**** | | 0.366 g | | 5.20% | | 11.86% | | 1.59% | | -2.69% | |  |
|  | *organ/body* | 0.09% | -0.52% | -10.01% | -14.66% | | **-25.80%*** | | 0.12% | | 6.36% | | 17.82% | | 10.19% | | 12.12% | |  |
|  | *organ/brain* | 25.38% | 3.50% | -13.25% | -24.06% | | -32.76% | | 18.59% | | 7.40% | | 14.58% | | 8.54% | | 1.56% | |  |
| testes | *mean weight* | 3.746 g | -12.01% | -4.28% | 0.40% | | -1.43% | | - | | - | | - | | - | | - | |  |
|  | *organ/body* | 0.59% | -14.84% | 3.96% | **+17.55%**** | | **+18.44%*** | | - | | - | | - | | - | | - | |  |
|  | *organ/brain* | 173.21% | -10.38% | -0.51% | 3.61% | | 2.59% | | - | | - | | - | | - | | - | |  |
| kidneys | *mean weight* | 3.933 g | 3.39% | 9.49% | **+13.52%*** | | **+12.51%*** | | 2.240 g | | -0.50% | | 4.28% | | 9.62% | | 6.08% | |  |
|  | *organ/body* | 0.62% | 1.51% | **+18.74%**** | **+32.59%**** | | **+32.33%**** | | 0.70% | | -2.49% | | 5.61% | | **+17.84%**** | | **+13.57%*** | |  |
|  | *organ/brain* | 181.91% | 5.43% | 13.63% | 17.25% | | 17.00% | | 113.42% | | 1.55% | | 6.61% | | 17.20% | | 10.89% | |  |
| pituitary gland | *mean weight* | 13.436 mg | 0.58% | -0.44% | -7.50% | | 3.51% | | 17.013 mg | | -5.53% | | -2.82% | | -16.63% | | -10.05% | |  |
|  | *organ/body* | 0.00% | -1.90% | 8.21% | 8.14% | | **+24.77%**** | | 0.005 | | 0.54% | | 5.94% | | -2.11% | | 2.75% | |  |
|  | *organ/brain* | 0.62% | 2.85% | 3.58% | -3.87% | | 7.97% | | 0.86 | | -3.27% | | -0.73% | | -11.10% | | -5.99% | |  |
| liver | *mean weight* | 16.218 g | 0.42% | -3.74% | -1.56% | | -4.98% | | 7.929 g | | 1.87% | | 6.28% | | 6.70% | | 3.34% | |  |
|  | *organ/body* | 2.56% | -1.91% | 3.96% | **+14.13%**** | | **+10.66%*** | | 2.47% | | 4.07% | | 12.08% | | **+19.00%**** | | **+13.54%*** | |  |
|  | *organ/brain* | 749.22% | 2.49% | 0.13% | 1.25% | | -1.15% | | 400.06% | | 4.35% | | 8.92% | | 14.46% | | 8.60% | |  |
| thyroid + parathyroid | *mean weight* | 35.553 mg | -8.70% | **-14.68%**** | -2.04% | | -2.53% | | 32.951 mg | | **-16.33%**** | | **-19.07%**** | | -2.11% | | 0.65% | |  |
|  | *organ/body* | 0.01% | -10.78% | -7.78% | 14.60% | | 14.51% | | 0.01% | | -18.68% | | -19.09% | | 3.76% | | 8.91% | |  |
|  | *organ/brain* | 1.64% | -6.71% | -11.19% | 0.94% | | 1.64% | | 1.68% | | -14.88% | | -17.90% | | 4.08% | | 4.77% | |  |
